# Dimensions of English language learner autonomy assessment: a systematic review of what is there and what is missing

**DOI:** 10.3389/fpsyg.2026.1711599

**Published:** 2026-02-11

**Authors:** Aiju Liu, Amelia Abdullah, Hong Dong

**Affiliations:** 1 English Teaching Department, Shandong College of Electronic Technology, Jinan, China; 2 School of Educational Studies, Universiti Sains Malaysia, Minden Heights, Malaysia

**Keywords:** assessment, dimensions, LAELL, PRISMA, purposes

## Abstract

Extensive research highlights the importance of learner autonomy in English language acquisition. Paradoxically, existing assessments for Learner Autonomy in English Language Learning (LAELL) narrowly focus on formal learning environments. This systematic mapping review, identifies frequently used dimensions, and highlights gaps in LAELL assessments within evolving learning contexts. Adhering to PRISMA guidelines, we identified 30 studies (1996–2025) across five databases. Key findings reveal that (1) *meta-cognitive dimension* is the most frequently addressed in the reviewed studies, followed by *motivation* and *social dimension*, while *technology*, *willingness*, *affective factors*, *critical thinking, political factors,* and *self-efficacy* are least presented; and (2) adapted questionnaires predominantly originated from older frameworks (50% from 1978–2009), with only 10% since 2016. Three gaps emerge: (1) overreliance on outdated questionnaire adaptations; (2) absence of LAELL assessments designed for informal learning contexts; and (3) insufficient attention to digital competences, particularly technology literacy and critical thinking. These findings highlight a potential misalignment between current assessment tools and contemporary learning environments. We suggest that LAELL assessments be updated to include dimensions of digital competence and critical thinking, and that skill-specific assessments be developed for productive domains like writing.

## Introduction

Research on learner autonomy has evolved over the past forty years (Holec, 1981; Stringer, 2024). It is commonly defined as a *capacity* (Little, 1996) to take control of one’s learning, which can manifest in varying degrees (Nunan, 1997). Traditionally conceptualized within formal education, this construct is being fundamentally reshaped by informal digital learning (Sockett, 2023; Zhang and Liu, 2024). Sockett and Toffoli (2012), for example, describe learner autonomy as the capacity to self-direct learning through the intentional use of online tools and resources beyond the classroom. This expansion necessitates a critical re-examination of how learner autonomy is assessed across diverse learning environments.

Given this shift, the role of assessment becomes critically important. Researchers highlight that assessment not only helps learners develop autonomy (Chong and Reinders, 2022) but also assists teachers in identifying its key dimensions and adapting instruction to diverse contexts (Lamb and Little, 2016). Considering English’s role as a lingua franca in politics, economics, and academia (Almusharraf, 2018), this review specifically examines Learner Autonomy in English Language Learning (LAELL). While numerous assessment tools for LAELL exist, they have primarily targeted formal learning contexts (Banat, 2022; Shen et al., 2020). This presents a critical research gap: the growing prevalence of informal digital learning creates a mismatch where existing assessments may not adequately capture the dimensions of autonomy relevant to the contemporary contexts. A systematic analysis of what is and is not being measured is therefore needed. Despite a growing body of research on learner autonomy, no systematic review has analyzed the dimensions underpinning LAELL assessments to identify this gap. The sole existing review (Chong and Reinders, 2022) identified commonly used assessment tools for fostering LAELL, offering valuable methodological insights but ignoring the underlying dimensions of autonomy. Building on their work, this systematic mapping review aims to examine autonomy dimensions within evolving learning environments.

LAELL is a context-dependent and multifaceted construct, comprising political, psychological, sociocultural, and technological dimensions (Oussou, 2024; Stringer, 2024). These dimensions dynamically respond to evolving educational contexts, with digital competence emerging as particularly critical within technology-mediated learning contexts (Chen and Liu, 2024). This focus on digital competence aligns with contemporary learners. The initial label *digital natives* (Evans and Robertson, 2020; Prensky, 2001a) has been largely supplanted by *digital learners* (Gallardo-Echenique et al., 2015), as the former is criticized for lacking empirical evidence and for inaccurately assuming younger generations possess high digital competence (Gallardo-Echenique et al., 2015).

Today, learning is increasingly shaped by digital environments, highlighting digital competence as a vital lifelong skill (Council of Europe, 2018). Supporting this, China’s CNNIC (2023) reports that over 80% of Generation Z (ages 20–29) possess basic digital competence. These findings suggest that learners raised with constant access to digital tools and social media tend to prefer technology-mediated learning over traditional methods (Sockett, 2023). This shift also demands stronger critical thinking skills to manage excessive information online (Bawden and Robinson, 2020). Consequently, there is a pressing need for research into whether existing LAELL assessments adequately address the characteristics of digital learners.

To address this gap, this review analyzed articles from 1996 to 2025 regarding current trends, dimensions, and gaps of LAELL assessments. The findings aim to provide insights for researchers and educators seeking to adopt, adapt, or develop innovative LAELL assessments suited for contemporary digital settings, ultimately fostering learners to develop learner autonomy. Specifically, the study seeks to answer the research questions outlined in Table 1.

**Table 1:** Research questions.

S. no	Research questions	Focus
1	What are the current trends of LAELL assessments?	Country, year, sampling, assessment types, research domains
2	What are the dimensions frequently used in LAELL assessments?	Dimensions of LAELL assessments, LAELL assessment purposes
3	What gaps are identified from the existing LAELL assessments?	Dimensions underrepresented in informal digital learning contexts

## Literature review

Research consistently highlights that LAELL is a dynamic and evolving concept (Stringer, 2024; Tassinari, 2012). During the era dominated by classroom-based education, learner autonomy was often perceived as an inborn yet teachable ability, primarily developed through in-class practices (Almusharraf, 2018). Nevertheless, the digital revolution has dramatically shifted LAELL research from in-class settings to informal digital learning contexts (Borges, 2022). Consequently, discussions on LAELL have increasingly extended to informal learning contexts, reflected in terms such as “*online informal learning of English*” (Toffoli and Sockett, 2015), “*out-of-class autonomous language learning*” (Lai, 2019), “*extramural English*” (Sundqvist and Sylvén, 2016), “*the digital wilds*” (Sockett, 2023), and “*informal digital learning of English (IDLE)*” (Zhang and Liu, 2024). This evolution reflects a transition in LAELL from teacher-dependent to self-directed learning, particularly evident in the rise of IDLE.

LAELL is inherently multidimensional, shaped by personal and contextual dimensions. Grounded in Benson’s (1997) three-dimensional framework (technical, psychological, and political), subsequent dimensions are progressively expanded (Gholami, 2016; Shen et al., 2020). This aligns with Benson’s (2001) statement, *“a multidimensional capacity that will take different forms for different individuals, and even for the same individual in different contexts or at different times”* (p. 47). In 2022, Borges further advanced this understanding by proposing a Complex Dynamic Model of Autonomy Development, including dimensions such as reflection, planning, and evaluation, autonomy support, and nested subsystems (e.g., motivation, identity, beliefs, and affective), as well as the broader learning context.

Despite this expansion, key dimensions essential for digital learning remain theoretically underdeveloped. While technology has traditionally been treated as an external tool or context in EFL learning (Enayati and Gilakjani, 2020; Chen, 2021), post-digital scholars argue it should be reconceived as a critical dimension that actively reshapes the cognitive, social, and affective foundations of learner autonomy (Campbell and Olteanu, 2024; Knox, 2019). Similarly, critical thinking in digital contexts extends beyond information evaluation to include an understanding of algorithm-driven content curation, platform biases, and the political economy of digital tools (Bawden and Robinson, 2020; Facione, 2000). These two dimensions are seldom integrated into LAELL assessment frameworks.

LAELL is not an *“all-or-nothing”* concept (Nunan, 1997, p. 92) but rather a complex, layered construct. LAELL is recognized as measurable, offering insights into learners’ progress. LAELL assessments serve multiple purposes, so that they can adapt to evolving learning environments, the traits of digital learners, and ongoing technology advancements. As such, research on LAELL assessments has expanded beyond measuring autonomy levels to examining readiness for LAELL (Kartal and Balçikanli, 2019; Oussou, 2024), and learners’ self-perceptions of autonomy (Hoa et al., 2019).

Despite the critical need for a comprehensive review of LAELL assessments, existing reviews remain scarce. The sole existing scoping review (Chong and Reinders, 2022) examined 61 articles, identifying assessment instruments such as questionnaires (*n =* 56), language tests (*n =* 19), interviews (*n =* 11), language tasks (*n =* 11), field notes (*n =* 2), and documents (*n =* 1). While this review categorized questionnaires into existing, adapted, and original types, it did not examine the evaluative dimensions used. To address this gap, our study provides a comprehensive analysis of dimensions in LAELL assessments, providing guidance for researchers developing innovative assessment tools.

## Materials and methods

### Research methodology and sources

We conducted a systematic mapping review for literature selection, analysis, and synthesis. This type of review is specifically designed to provide a broad overview of a research field rather than to answer a highly focused question (Dai and Ke, 2022). This approach is well-suited for outlining learner autonomy structure and identifying its dominant research trends, which aligns with the exploratory aims of our research questions. Following the Preferred Reporting Items for Systematic Review and Meta-Analysis (PRISMA) guideline, we applied its procedures throughout the study and reported the results. The search was performed across Scopus, Web of Science (WOS), Science Direct, ERIC, and supplemented by Google Scholar. This combination was essential for our systematic mapping review. While the former four databases ensure coverage of high-quality, peer-reviewed literature, the inclusion of Google Scholar expands the search to retrieve relevant grey literature and articles from a broader range of journals and conferences (Haddaway et al., 2015).

### Eligibility criteria and search strings

Inclusion and exclusion criteria were set prior to the literature selection (see Table 2). The criteria focused on database, language, timeframe, publication status, methodology used, and respondents. The decision to limit the review to English-language publications ensures consistency and rigor throughout the review process. While this is a common limitation, research indicates it has minimal impact on overall conclusions (Morrison et al., 2012). Moreover, the review covers studies published from 1996, when Warschauer first highlighted the importance of investigating computer-mediated learning for English language (Warschauer et al., 1996).

**Table 2:** The inclusion/exclusion criteria for the study.

Criteria	Inclusion	Exclusion
Language	English language	Articles not written in English language
Timeframe	1996-May 2025	Publications before 1996 or after 2025
Publication	peer-reviewed journal articles	Books, editorials, review papers, conference papers, dissertation theses, and retracted publications
Database	Scopus, WOS, ScienceDirect, ERIC, Google Scholar	Other databases
Discipline	Empirical studies	Articles other than empirical studies
Participants	Students	Non-students

Accurate keywords as well as their synonyms and variations were identified to retrieve more relevant documents (Grames et al., 2019). Boolean operators (i.e., AND, OR, NOT) and wildcard (i.e., an asterisk) were included in the search strings. While many studies use “factors,” “facets,” and “aspects” to examine perceptions or correlations related to learner autonomy (Fotiadou et al., 2017; Basri, 2023), we specifically targeted “dimension*” and “construct*” to ensure our assessment-focused scope. Meantime, related terms such as “learner agency” and “self-directed learning” were excluded from the search strings. While closely related, learner autonomy specifically concerns *how* learning is controlled, whereas agency addresses the *why* and *who* behind learning behavior, and self-directed learning often implies a broader educational context beyond language learning (Rasulova and Ottoson, 2022). This exclusion ensured that the review remained focused on instruments explicitly designed to assess LAELL. As such, the search string for Scopus, WOS, and ERIC was: (“learner autonomy” OR autonomous) AND English AND (dimension* OR construct*) AND (questionnaire* OR instrument OR assessment OR evaluation OR measure* OR rubric OR scale) in the title and abstract of papers. Due to the maximum of eight Boolean words for Science Direct and Google Scholar, the search string was: [“learner autonomy” AND English AND (dimension OR construct) AND (questionnaire OR assessment OR evaluation OR measure)].

To manage the high volume generated from Google Scholar, the search was limited to the first 100 records ranked by “relevance,” a metric determined by Google Scholar’s ranking algorithm and citation count (Beel and Gipp, 2009; Haddaway et al., 2015). This screening threshold prioritizes records with greater scholarly impact and relevance to the query (Beel and Gipp, 2009). To ensure topical relevance of the retrieved items, all 100 results underwent manual screening against the study’s predefined inclusion/exclusion criteria (see Table 2). During this process, duplicates (against both internal Google Scholar results and records from other databases) were removed, and irrelevant items were excluded.

### Selection process

**Figure 1:**
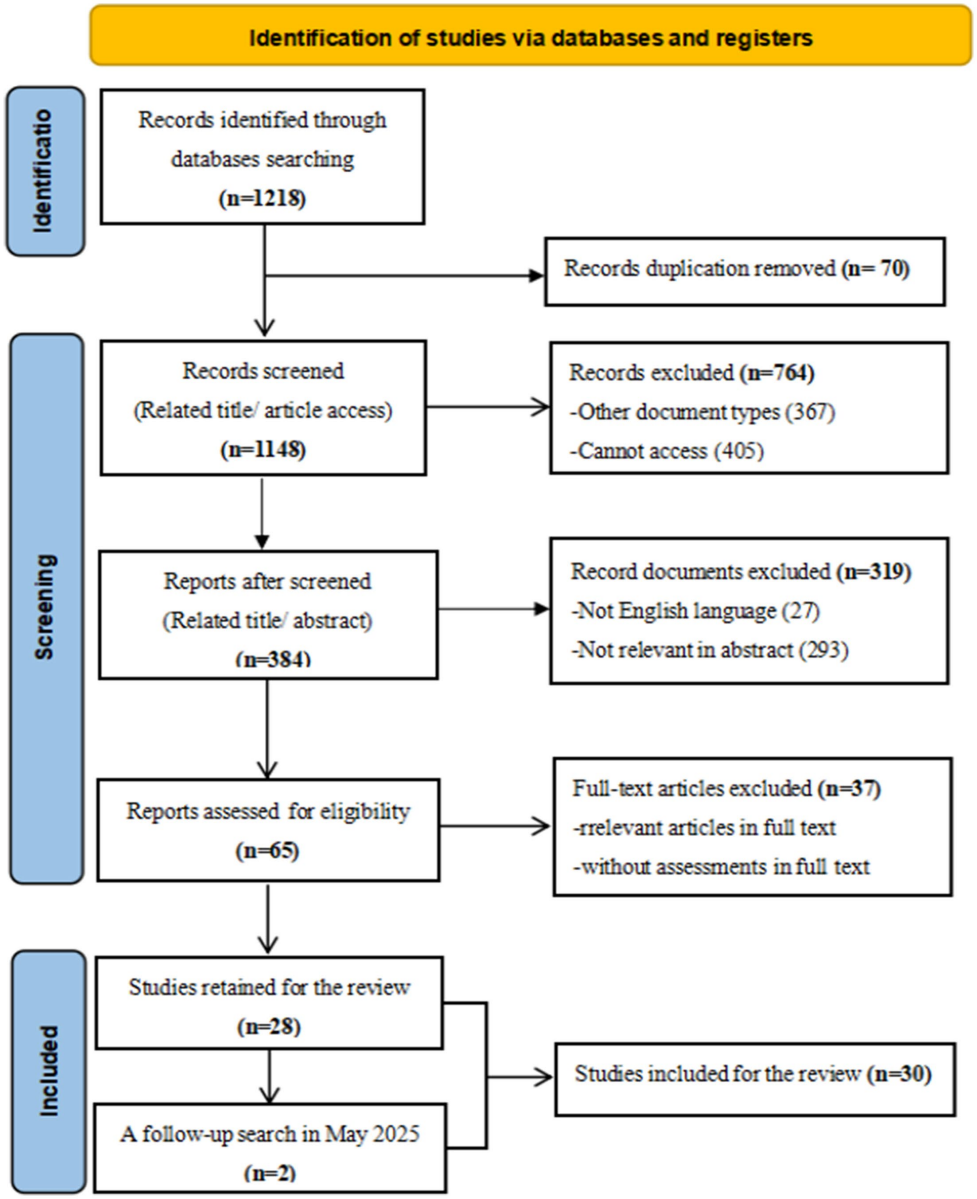
Flow diagram for systematic reviews (adapted from Page et al., 2021).

Manual searching: We conducted the first search following PRISMA guidelines (see Figure 1) on December 10, 2024. The initial search yielded 1,218 articles. After removing 70 duplicates and excluding 764 articles that did not meet the inclusion criteria, 384 articles remained. Two reviewers independently assessed their eligibility, and 319 articles were excluded. This left 65 studies for full-text screening, of which 37 were excluded due to insufficient information on assessments. Ultimately, 28 articles met the inclusion criteria. To ensure the review included the most recent research, a follow-up search was conducted on May 29, 2025, covering publications from December 2024 to May 2025 across the same five databases, which yielded 171 articles. After removing duplication and irrelevant studies, 2 articles were used for analysis. Altogether, 30 studies were analyzed for this review.

### Data collection process and quality assessment

To minimize bias and ensure reliability, we implemented a dual-review process for data collection and extraction (Pérez et al., 2020). Two reviewers (A and Z) independently extracted data using an electronic data extraction form, including the article title, abstract, reference details, country, discipline, sample size, distribution scope (e.g., national or international), methodology, reliability, and validity of the assessments. Following the inclusion criteria (see Table 2), each study underwent a quality appraisal: (1) alignment with research aims; (2) sampling justification; (3) instrument reliability; (4) instrument validity; and (5) trustworthiness of findings. Consistent with the objectives of a systematic mapping review, this assessment was used not to exclude studies, but to characterize the methodological robustness of the evidence base. This critical appraisal directly informed the interpretation of the findings, allowing for nuanced consideration of factors such as whether identified trends were supported by studies with small samples or instruments with limited validation. Inter-rater agreement for the eligibility screening was assessed using Cohen’s Kappa statistic (Pérez et al., 2020), and it was 0.700, indicating substantial agreement.

### Statistical analysis

To systematically identify and define the dimensions present in LAELL assessments, we employed a hybrid content analysis approach guided by a structured codebook. This process combined deductive and inductive reasoning. First, we developed a deductive framework based on established theoretical dimensions of learner autonomy: technical, psychological, political, social, and technological (Oussou, 2024; Stringer, 2024). These five domains formed the initial structure of the codebook.

Within each domain, we then conducted an inductive thematic analysis. Two reviewers independently coded the assessment items to identify recurring themes. Throughout this process, the codebook was iteratively refined to include emerging inductive themes, along with illustrative examples drawn from the data (see Table 3). Constant comparison was used to ensure themes were grounded in the data.

**Table 3:** Coding excerpts.

Dimension	Code	Sub-dimension	Example excepts
Psychological	A1	Motivation	*“Motivational sub-dimension signifies intrinsic and extrinsic motivation towards learning English.”* (Swatevacharkul and Boonma, 2021)
	A2	Confidence	*“Gaining confidence or raising self-efficacy again indicated elevated agency for learners, and a higher level of learner autonomy”* (Shen et al., 2020)
	A3	Willingness	*“Affective factors like willingness play an important role in the development of learner autonomy.”* (Van Nguyen and Habók, 2021)

Through reviewer consensus, the inductive thematic analysis yielded fourteen distinct dimensions. To ensure a faithful representation of the literature and maintain the granularity required for a precise mapping, we have preserved these as distinct constructs. While we acknowledge potential conceptual overlaps (e.g., between *affective factors* and *motivation*), this approach avoids prematurely collapsing the varied conceptualizations present in the source instruments. Consequently, our analysis is structured around a clear conceptual hierarchy: the five deductive domains represent overarching domains on autonomy (e.g., the Psychological domain), while the fourteen inductive dimensions are the specific constructs within them (e.g., *motivation*, *self-efficacy* within the Psychological domain).

### Validity and inter-rater reliability

To ensure the validity and accuracy of the coding process, two reviewers (A and Z) first independently coded a randomly selected 20% subset of the included studies. Inter-coder reliability was assessed using Cohen’s Kappa, yielding a value of 0.720, which indicates substantial agreement (McHugh, 2012). Given the complexity of autonomy constructs, coding disagreements were resolved through a structured consensus process. Reviewers jointly reexamined each conflicting item against the original source text and discussed interpretations until reaching a shared, evidence-based decision. This two-stage approach (i.e., independent coding followed by deliberative consensus) is particularly suited for research requiring high conceptual inference. It strengthens the methodological rigor of the coding framework, enhancing the robustness and credibility of the study’s findings.

## Results

RQ 1: What are the current trends in LAELL assessments?

We identified a growing research trend in LAELL assessments through our literature mapping, which analyzed the distribution of relevant studies by country, publication year, participant groups, research instruments, and assessment domains.

**Figure 2:**
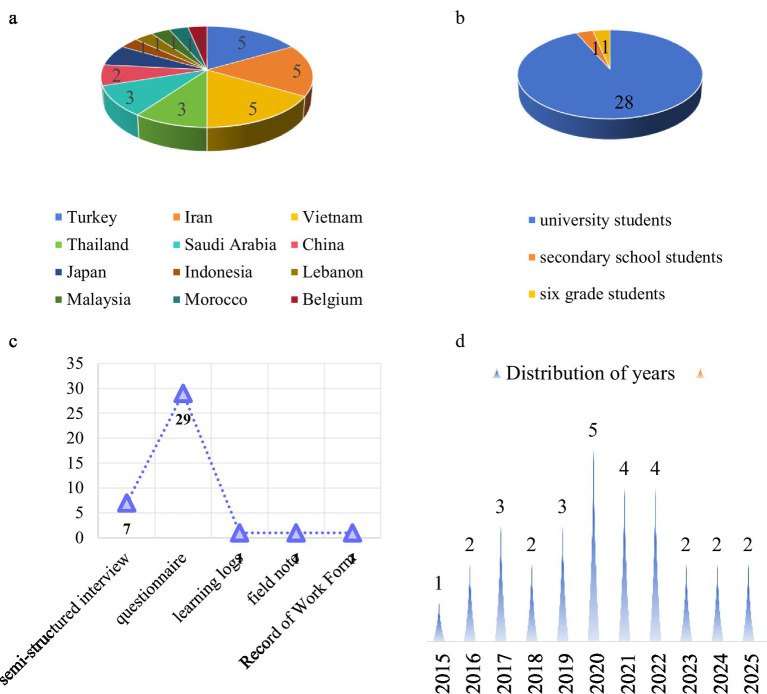
**(a)** Distribution of articles. **(b)** Composition of participants. **(c)** Research instruments. **(d)** Distribution of the year.

*Distribution of studies.* As shown in Figure 2a, the studies were distributed across multiple countries. Iran, Vietnam, and Turkey (*n =* 5 each) contributed the most studies, followed by Thailand and Saudi Arabia (*n =* 3 each), China and Japan (*n =* 2 each), Indonesia, Lebanon, Malaysia, Belgium, and Morocco (*n =* 1 each). Geographically, 28 articles originated from Asia, while one each came from Europe and Africa. This distribution should be interpreted within the constraints of our search strategy, which was limited to English-language publications using specific terminology (e.g., dimension, construct). Nevertheless, the distribution is noteworthy, as many Asian countries traditionally follow hierarchical and teacher-centered education systems (Leong et al., 2018). The growing focus on LAELL may suggest a shift toward more student-centered learning approaches in English language education across the region.

*Composition of participants.* The result aligns with the scoping review by Chong and Reinder (2022), indicating that 90% of the studies focused on higher education, with the remaining studies examining secondary schools and primary schools. As shown in Figure 2b, the predominance of university-level participants (93%) likely reflects key traits of contemporary tertiary learners: (1) developed metacognitive strategies, (2) stronger intrinsic motivation for autonomous learning, (3) greater sense of responsibility, (4) critical use of teacher/peer support, and (4) higher technology proficiency for self-directed learning via IDLE.

*Research instruments.* Figure 2c revealed that questionnaires (*n =* 29) were the most used research instrument in these articles, followed by semi-structured interviews (*n =* 7), field notes (*n =* 1), learning logs (*n =* 1), and records of work (*n =* 1). Quantitative instruments dominate the LAELL assessment studies. Notably, of the questionnaires used, twenty were adapted from prior works, five were adopted directly from existing questionnaires, and only three were originally developed.

*Distribution of year.* As illustrated in Figure 2d, there is a lack of assessment-related research prior to 2015, likely because early studies on learner autonomy primarily focused on its description and conceptualization. Despite annual LAELL assessments from 2015 to 2025, there has been no significant increase over the past decade.

**Figure 3:**
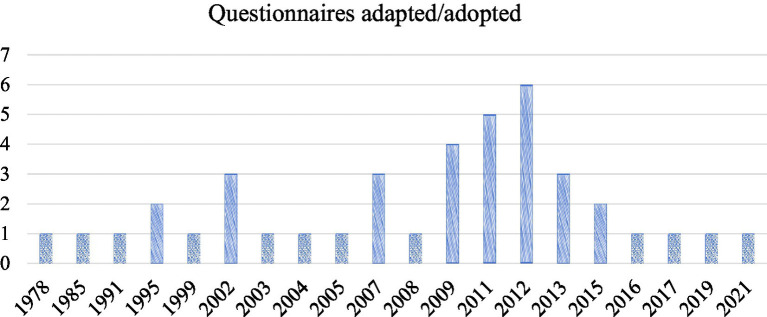
Questionnaire adapted presentation.

*Innovation deficit in Questionnaires.* Questionnaires adapted and adopted for LAELL assessment were predominantly from prior studies (*n =* 20). As Figure 3 demonstrates, their distribution over time is as follows: 1978–1999 (*n =* 6), 2002–2009 (*n =* 14), 2011–2015 (*n =* 16), and 2016–2021 (*n =* 4). This trend suggests many questionnaires currently in use were developed 10–20 years ago and have not been updated to reflect the evolving nature of LAELL and contemporary students.

*Research domain.* LAELL is widely acknowledged as essential for English language acquisition. However, as shown in Table 4, a large portion of LAELL-related research addressed English learning as a general concept rather than targeting specific language skills. Our systematic mapping review reveals 23 articles addressed general English learning; only a few focused on discrete skills: writing (*n =* 4), vocabulary (*n =* 2), and reading (*n =* 1).

RQ 2: What are evaluative dimensions frequently used for LAELL assessments?

**Table 4:** Distribution of studies by language skill focus.

Domain	References
General English	Ceylan (2015); Larsari and Oghli (2016); Orakci and Gelisli (2017); Alzubi et al. (2017); Ünal et al. (2017); Gamble et al. (2018); Ruelens (2019); Kartal and Balçikanli (2019); Hoa et al. (2019); Boonma and Swatevacharkul (2020); Pasaribu (2020); Swatevacharkul and Boonma (2020); Tuan (2021); Van Nguyen and Habók (2021); Ghobain and Zughaibi (2021); Baharom and Shaari (2022); Irgatoğlu et al. (2022); Chen (2022); Suwannaphim and Vibulphol (2023); Cao and Pho (2024); Oussou et al. (2024); Rezai and Goodarzi (2025); Phan and Huynh (2025)
Vocabulary	Sato et al. (2020); Almusharraf (2018)
Reading	Pasaribu (2020)
Writing	Banat (2022); Shen et al. (2020); Mohammadi Zenouzagh et al. (2023); Tajmirriahi and Rezvani (2021)

**Figure 4:**
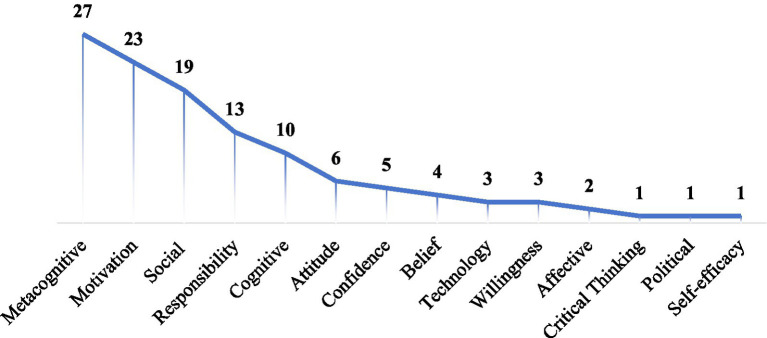
Prevalence of dimensions in LAELL assessments.

Through a hybrid coding process, fourteen dimensions of learner autonomy are presented in Figure 4 and detailed in the Appendix. It is important to note that these dimensions represent specific constructs derived from the assessment instruments reviewed, and they operate within a broader conceptual landscape. For instance, *motivation* and *self-efficacy* are psychological domain, while *cognitive* belongs to the technical domain. In terms of frequency, the *metacognitive dimension* was the most frequently represented, followed by *motivation* and the *social dimension*. In contrast, dimensions such as *technology*, *willingness*, *affective factors*, *critical thinking*, *political*, and *self-efficacy* were the least represented in the reviewed assessments.

**Figure 5:**
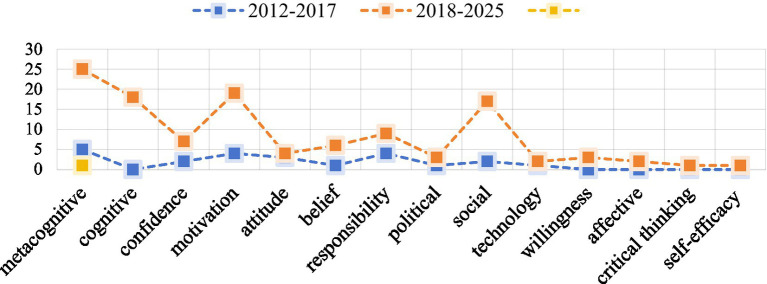
Comparison between two generation stages.

Furthermore, a temporal analysis indicates the emphasis on specific dimensions has shifted in response to evolving learner demographics. While *digital natives* span four generational stages (1996–2006, 2007–2011, 2012–2017, and 2018-present) (Evans and Robertson, 2020), LAELL assessments primarily focused on the latter two stages (2012–2017 and 2018–2025). These two stages show differences in their evaluative dimensions. As demonstrated in Figure 5, the third stage (2012 to 2017) emphasized *metacognitive* (*n =* 5), *motivation* (*n =* 4), and *responsibility* dimensions (*n =* 4), while the fourth stage (2018 to 2025) emphasized *metacognitive* (*n =* 22), *motivation* (*n =* 19), *cognitive* (*n =* 8), and *social dimension* (*n =* 17). This aligns with Uslu and Durak’s (2022) findings that *meta-cognitive* and *motivation* dimensions are strong predictors of learner autonomy. Besides, the increased attention to *social dimension* reflects the importance of assessing peer and teacher reliance. Other psychological dimensions such as *confidence*, *belief*, *attitude*, *willingness*, and *affective* were also taken into the LAELL assessments. Notably, competences essential for informal digital learning, particularly *critical thinking* and *technology*, were least represented.

In addition, the reviewed assessments indicate that these dimensions serve diverse evaluative purposes. As outlined in the Appendix, these assessments were used to measure LAELL levels (*n =* 22), learners’ perceptions of LAELL (*n =* 3), readiness for LAELL (*n =* 3), psychological dimension of LAELL (*n =* 1), and meta-cognitive dimension of LAELL (*n =* 1).

RQ 3: What gaps are identified from the existing LAELL assessments?

Existing research reveals four key gaps in LAELL assessment: (1) region and demography, (2) learning environments, (3) temporal factors (date/timeliness), (4) language skills, and (5) methodological shortcomings.

First, overrepresentation of Asian higher education contexts (90% of participants) limits cross-cultural generalizability. While adult learners often exhibit stronger metacognitive skills and higher motivation, this narrow focus in the literature we analyzed overlooks valuable insights from younger learners from primary and secondary levels. Future research should expand the demographic scope to provide a more comprehensive understanding of LAELL across cultural contexts and educational levels.

Second, existing LAELL assessments largely overlook informal digital learning settings. Research confirms that LAELL is dynamic and multidimensional (Benson, 2001; Nunan, 1997), with recent research on LAELL gradually transforming focus from formal to informal learning contexts (Rezai and Goodarzi, 2025). However, studies predominantly examine formal settings (*n =* 19, including 3 for blended learning), with only one for informal learning contexts. This is a striking imbalance given the growing prevalence of informal digital learning environments.

Third, LAELL assessments are mostly outdated. Most instruments relied on outdated questionnaires (10–20 years ago). For example, despite a recent study by Rezai and Goodarzi (2025) that adapted a questionnaire to assess LAELL through IDLE, all dimensions were adapted from Barnard et al. (2009). Of the studies reviewed, dimensions mostly adapted or adopted from prior studies include meta-cognition and motivation. In contrast, emerging dimensions such as critical thinking and technology competence (Tan et al., 2023) are underrepresented. Our review reveals that few studies realized the need to include updated dimensions except for Ruelens (2019), who expanded the metacognitive dimension to include “*transferring skills across contexts*.” Furthermore, existing assessments also overlook technology dimensions crucial to IDLE. Since the concept of *“digital natives”* was introduced in 1996, technology has profoundly influenced how individuals learn foreign languages (Evans and Robertson, 2020; Prensky, 2001a). The oversight of technological dimensions limits the reliability of LAELL assessments in the technology-driven context (Chen and Liu, 2024). These findings suggest a gap that LAELL assessment instruments should either be newly developed or adapt existing instruments with contemporary dimensions.

Fourth, LAELL assessments mostly focused on general English (77% of the studies), with limited attention to specific skills such as vocabulary, reading, and writing. However, English language learning generally refers to listening, speaking, reading, and writing. Among these, speaking and writing (output skills) are particularly challenging due to their active production demands (Shen et al., 2020; Aziz and Kashinathan, 2021). Future research should assess current LAELL levels in these output skills and explore strategies for improvement.

Last but not least, quantitative tools such as questionnaires dominate LAELL assessments (as shown in Appendix), but they often lack the depth required to fully understand the subjective experiences of learners. Triangulation, which involves using multiple qualitative research methods such as interviews, focus groups, or case studies, would allow for a deeper exploration of the subjective experiences of learners, as well as the contextual factors influencing their autonomy (Carter, 2014). These methods could provide richer insights into the affective and cognitive dimensions of LAELL that are often difficult to capture through quantitative surveys.

## Discussion

This systematic review highlights a critical misalignment between contemporary language learning realities and the dimensions measured by prevailing LAELL assessments. A primary issue is the over-reliance on outdated frameworks. Approximately 70% of existing tools we analyzed are adapted from pre-2015 models designed for formal classrooms. Consequently, they prioritize dimensions like metacognition and teacher support, which are rooted in formal education contexts (Little, 1996), while largely overlooking the critical role of technology literacy in informal learning (Benson, 2005). As a result, these assessments fail to account for the autonomous learning in informal digital environments. This discrepancy stems from a longstanding research bias toward formal settings. Despite robust evidence affirming the prevalence and efficacy of IDLE (Sockett, 2023; Zhang and Liu, 2024), it is frequently treated as an experimental variable in research rather than a well-established mode of EFL acquisition (He and Zhu, 2017). This has led to a notable scarcity of assessments designed for IDLE contexts.

The dimensional focus of existing LAELL assessments reveals a critical concern. While established psychological factors like motivation and self-regulation remain central (Banat, 2022; Benson, 2011; Deci and Ryan, 2000), emerging competencies essential for learning *“in the digital wild”* (Han and Reinhardt, 2022) are underrepresented. For example, both technology literacy (*n =* 3) and critical thinking skill (*n =* 1) are rarely assessed, despite the former being a prerequisite for IDLE (Reinders et al., 2022) and the latter involving *“reflectively making sound judgments”* (Facione, 2000).

A further imbalance is evident in the linguistic scope of LAELL research. Most assessments (77%) target general English proficiency, with limited attention to discrete language skills such as listening, speaking, reading, or writing (Shen et al., 2020). It is problematic given that productive skills like speaking and writing are especially challenging due to their generative nature (Aziz and Kashinathan, 2021; Shen et al., 2020). Therefore, future research should prioritize developing assessments of learner autonomy within these specific output domains.

The review also indicates an evolution in the purpose of LAELL assessments. Moving beyond merely measuring autonomous behaviors, contemporary tools increasingly aim to diagnose learners’ psychological states, including their perceptions, readiness, and beliefs about autonomous learning (Chan, 2001; Hoa et al., 2019). This trend reflects a growing consensus that autonomy is not merely a behavioral output but is fundamentally mediated by cognitive and affective factors (Feng and Yang, 2025). By probing these dimensions, assessments can reveal not just *whether* learners are autonomous, but also *how* and *why* they engage with EFL learning across diverse environments.

While the hybrid deductive-inductive approach allowed us to map the LAELL comprehensively, the resulting fourteen dimensions exhibit varying levels of conceptual clarity and overlap. For instance, *affective factors* and *willingness* show substantial theoretical kinship with core psychological constructs like *motivation*, suggesting potential for regrouping under broader, more parsimonious categories (e.g., “Affective Dimensions”). Future research and assessment development would benefit from a more streamlined and theoretically unified framework that clearly distinguishes between enduring learner traits (e.g., *self-efficacy*), teachable skills (e.g., *metacognitive strategies, critical thinking*), and contextual enablers (e.g., access to technology, social support). Such refinement would improve the precision, comparability, and practical utility of LAELL assessments.

Finally, the geographical concentration of the reviewed studies in Asia represents both a strength and a limitation. While it provides region-specific insights, it also introduces a regional and cultural bias. The predominance of research from historically teacher-centered educational contexts may overemphasis metacognition, which relate to gaining independence from instructor-led structures. This perspective might overlook the understandings of autonomy prevalent in learner-centered educational cultures.

## Conclusion

This systematic review synthesized 30 studies to map the dimensions, trends, and gaps in LAELL assessments. The findings reveal three critical issues: (1) a heavy reliance on assessment tools adapted from outdated frameworks, (2) a predominant focus on formal learning contexts, and (3) limited attention to dimensions essential for digital learning such as *digital competence* and *critical thinking*. To align assessment with the reality of contemporary EFL learning, we propose four key recommendations for research and practice.

Our first recommendation is to modernize LAELL assessment tools. Given the finding that approximately 70% of instruments are adapted from pre-2015 models, we recommend to update or newly develop LAELL assessments. This consideration is critical because current instruments were primarily designed for pre-digital learners, whereas today’s learners rely heavily on digital technology for informal language acquisition. The adaptation of traditional dimensions for digital environments is necessary, which aligns with Ruelens (2019) who demonstrates *“skill transfer across contexts.”* The second recommendation is developing skill-specific LAELL scales. Our finding showed that 77% of the reviewed assessments targeted general English proficiency, with productive skills being underrepresented (Banat, 2022; Shen et al., 2020). Therefore, we recommend the development of skill-specific LAELL scales. This would address the imbalance in the research and provide more nuanced insights into how autonomy manifests in different linguistic domains. Responding to the underrepresentation of digital competence and critical thinking in LAELL assessments, we recommend that educators explicitly integrate these skills into EFL curricula. This could involve modules on “learning how to be a critical digital learner,” and “providing students strategies for IDLE.” Besides, educators could further investigate the correlation between specific dimensions and IDLE performance using reflective journals or interviews. Finally, the methodological patterns observed in the review indicate a predominance of quantitative surveys. To gain a deep understanding of LAELL, future studies could prioritize mixed-methods and longitudinal designs. Combining quantitative scales or tests with qualitative methods like interviews, reflective journals, and learning logs to capture the complexity of LAELL in digital environments. While quantitative surveys dominate the current studies (see Figure 2), qualitative approaches can reveal learners’ subjective experiences, track their autonomy development, and document informal learning practices.

The study has two primary limitations. First, we excluded terms such as “learner agency” or “self-directed learning” to maintain conceptual precision, as these represent distinct theoretical frameworks despite frequent conflation with learner autonomy. This exclusion may explain the pronounced Asian focus in our findings, since European researchers frequently use these alternative terms. Second, database coverage was limited to English-language publications (Scopus, WOS, ScienceDirect, ERIC, and Google Scholar), omitting relevant non-English studies. For instance, although learner autonomy is emphasized in China, studies in Chinese databases such as CNKI were excluded (Farivar and Rahimi, 2015). Future research should expand search terms and include multilingual databases to ensure a more comprehensive coverage of LAELL assessments.

## Data Availability

The original contributions presented in the study are included in the article/Supplementary material, further inquiries can be directed to the corresponding author.
