# Supplementary material for: Dimensions of English language learner autonomy assessment: a systematic review of what is there and what is missing
Source: Front Psychol. 2026 Feb 11;17:1711599. doi: 10.3389/fpsyg.2026.1711599 (PMC12932582; doi:10.3389/fpsyg.2026.1711599)
Supplement: Supplementary file 1 [file Table_1.docx]

Appendix. LAELL Assessment Descriptions & Dimensions

| Note: Cognitive: *Cog*; Meta-cognitive: *Meta-c*; Confidence: *Con*; Motivation: *Mot*; Critical thinking: *Cri-th*; Attitude: *Att*; Belief: *Be*; Responsibility:*Resp*; Affective: *Aff* Willingness: *Will*; Self-efficacy: *Se-eff;* university students: *USt* | | | | | | | | | | | | | | | | | |
| --- | --- | --- | --- | --- | --- | --- | --- | --- | --- | --- | --- | --- | --- | --- | --- | --- | --- |
| Article | *****Assessment instrument (**AI**)  *****Assessment types (AT)  (existing/ original/ adapted)  *****Assessment purposes (**AP**)  *****Assessment Setting (**AS**) | Dimensions | | | | | | | | | | | | | | Validity & reliability | Sample |
|  |  | technical | | psychological | | | | | | | | | political | social | technology |  |  |
|  |  | *Cog* | *Meta-c* | *Con* | *Mot* | *Cri-th* | *Att* | *Be* | *Resp* | *Aff* | *Will* | *Se-eff* |  |  |  |  |  |
| Rezai and Goodarzi (2025) | **AI:** questionnaire  **AT:** adapted from Barnard et al. (2009)  **AP:** assess LA in IDLE  **AS:** outside the classroom | √ | √ |  |  |  |  |  |  |  |  |  |  |  |  | α = 0.75 | 325 *USt* |
| Phan and Huynh (2025) | **AI:** questionnaire  **AT:** adapted from Nguyen and Habók’s (2021)  **AP:** perceptions of LA level  **AS:** inside the classroom |  | √ |  | √ |  |  |  |  |  | √ |  |  | √ |  | α =0.7 | 334 *USt* |
| Oussou (2024) | **AI:** questionnaire  **AT:** adapted from Knowles et al. (2015), Phan (2021), Chan et al. (2010), Larsen-Freeman et al. (2021), Cotterall (1999), Macaskill and Taylor (2010), Tassinari (2012), Ruelens (2019), Shen et al. (2020)  **AP:** assess LA level  **AS:** in/outside the classroom | √ | √ |  |  |  |  | √ | √ |  |  |  |  | √ |  | α = 0.80 | 245 *USt* |
| Cao and Pho (2024) | **AI:** questionnaire; interview  **AT:** adapted from Nguyen (2012) , Macaskill and Taylor (2010), Ruelens (2019)  **AP:** assess LA level  **AS:** in/outside the classroom |  | √ |  | √ |  | √ |  |  |  |  |  |  |  |  | α > 0.7 | 220 *USt* |
| Zenouzagh et al. (2023) | **AI:** questionnaire  **AT:** existing questionnaire from Nguyen and Habók (2021)  **AP:** assess LA level  **AS:** formal online learning |  | √ |  | √ |  |  | √ |  |  |  |  |  |  |  | 0.76 < α < 0.91 | 40 *USt* |
| Suwannaphim and Vibulphol (2023) | **AI:** questionnaire; interview; learning logs  **AT:** adapted from Murase (2015)  **AP:** assess LA level  **AS:** inside the classroom | √ | √ |  | √ |  |  |  |  |  |  |  | √ | √ |  | α = 0.98 | 39 secondary students |
| Chen (2022) | **AI:** questionnaire  **AT:** adapted from Cotterall (1995), Heigham and Croker (2009), Creswell (2009), Alrabai (2017)  **AP:** assess LA level  **AS:** blended learning |  | √ |  | √ |  |  |  | √ |  | √ |  |  |  |  | 0.75 < α < 0.88 | 120 *USt* |
| Baharom  and Shaari (2022) | **AI:** field note; interview  **AT:** adapted from Alabdelwahab (2002)  **AP:** assess psychological dimension of LA  **AS:** inside the classroom |  |  | √ | **√** |  |  |  |  | √ |  |  |  | √ |  | / | 2 *USt* |
| Irgatoğlu et al. (2022) | **AI:** questionnaire  **AT:** adapted from Zhang and Li (2004)  **AP:** assess LA level  **AS:** inside the classroom |  |  | √ | **√** |  |  |  |  |  | **√** |  |  |  |  | α = 0.73 | 155 *USt* |
| Banat (2022) | **AI:** questionnaire  record of work Form  **AT:** adapted from Naiman et  al. (1978), Candy (1991)  **AP:** assess meta-cognitive dimension of LA  **AS:** inside the classroom |  | √ |  | √ |  | √ | √ |  |  |  |  |  | √ |  | α = 0.823 | 39 *USt* |
| Nguyen and Habók (2021) | **AI:** questionnaire  **AT:** adapted from Chan et al. (2002), Cotterall (1999), Le (2013), Swatevacharkul (2009), Hsu (2005)  **AP:** assess LA level  **AS:** in/outside the classroom |  | √ |  | √ |  |  |  |  |  |  |  |  | √ |  | α = 0.902 | 1565 *USt* |
| Ghobain and Zughaibi (2021) | **AI:** questionnaire  **AT:** existing questionnaire from Joshi (2011)  **AP:** assess readiness for LA  **AS:** in/outside the classroom |  | √ | √ | √ |  |  |  | √ |  |  |  |  | √ | √ | α =0.84 | 802 *USt* |
| Tajmirriahi and Rezvani (2021) | **AI:** questionnaire  **AT:** adapted from Yeung (2016), Cottrell (1995)  **AP:** measure LA level  **AS:** inside the classroom | √ | √ |  | √ |  | √ |  |  |  |  | √ |  | √ |  | α =0.817 | 69 *USt* |
| Tuan (2021) | **AI:** questionnaire; interview  **AT:** adapted from Spratt et al. (2015), Borg and Al-Busaidi (2012)  **AP:** assess readiness for LA  **AS:** in/outside the classroom | √ | √ |  |  |  |  |  | √ |  |  |  |  | √ |  | α =0.89 | 50 *USt* |
| Sato et al. (2020) | **AI:** questionnaire  **AT:** adapted from Benson (1997), Dang (2012), Oxford (2003), Pennycook (1997)  **AP:** assess LA level  **AS:** blended learning |  | √ |  | √ |  |  |  |  |  |  |  |  |  |  | α = 0.936 | 94 *USt* |
| Boonma and Swatevacharkul (2020) | **AI:** questionnaire  **AT:** adapted from Cohen, Oxford, and Chi (2002)  **AP:** assess LA level  **AS:** inside the classroom | √ | √ | √ | √ | √ |  |  |  | √ |  |  | √ | √ |  | α = 0.95 | 226 *USt* |
| Swatevacharkul and Boonma (2020) | **AI:** questionnaire  **AT:** adapted from Murase (2015)  **AP:** assess LA level  **AS:** inside the classroom | √ | √ |  | √ |  |  |  |  |  |  |  | √ | √ |  | α = 0.94 | 19 *USt* |
| Pasaribu (2020) | **AI:** questionnaire  **AT:** adapted from Allen (2013) and Dam (2011)  **AP:** assess LA level  **AS:** blended learning |  | √ | √ | √ |  | √ |  | √ |  |  |  |  | √ | √ | / | 25 *USt* |
| Shen et al. (2020) | **AI:** questionnaire  **AT:** adapted from Cotterall (1995), Dixon (2011)  **AP:** assess LA level  **AS:** inside the classroom |  | √ | √ |  |  |  | √ | √ |  |  |  |  | √ |  | α = 0.799 | 70 *USt* |
| Ruelens (2019) | **AI:** questionnaire  **AT:** original  **AP:** assess LA level  **AS:** inside the classroom | √ | √ | √ | √ |  |  | √ |  |  |  | √ |  | √ |  | 0.76 < α < 0.90 | 63 *USt* |
| Kartal and Balçikanli (2019) | **AI:** questionnaire; interview  **AT:** existing questionnaire from Karabıyık (2008)  **AP:** assess readiness for LA  **AS:** in/outside the classroom |  | √ |  | √ |  |  |  | √ |  |  |  |  | √ |  | / | 110 *USt* |
| Hoa et al. (2019) | **AI:** questionnaire; interview  **AT:** original  **AP:** self-perception  **AS:** in/outside the classroom |  | √ |  |  |  |  |  | √ |  |  |  |  | √ |  | α > 0.700 | 80 *USt* |
| Almusharraf (2018) | **AI:** interview (case study)  **AT:** guided by Dickinson (1993) and Benson (2007)  **AP:** assess LA level  **AS:** inside the classroom |  | √ |  | √ |  |  | √ |  |  |  |  |  | √ |  | / | 6 *USt* |
| Gamble et al. (2018) | **AI:** questionnaire  **AT:** adapted Chan et al. (2002), Deci (1995), Deci and Ryan (1985)  **AP:** assess LA level  **AS:** in/outside the classroom |  | √ |  | √ |  |  |  | √ |  |  |  |  |  |  | / | 958 *USt* |
| Orakci and Gelisli (2017) | **AI:** questionnaire  **AT:** original  **AP:** assess LA level  **AS:** inside the classroom |  | √ |  | √ |  |  |  | √ |  |  |  |  |  |  | α = 0.965 | 297 6th grade |
| Alzubi and Pandian (2017) | **AI:** questionnaire  **AT:** existing questionnaire from Dixon (2011)  **AP:** assess LA level  **AS:** in/outside the classroom | √ | √ | √ | √ |  | √ |  | √ |  |  |  |  |  | √ | 0.61 < α < 0.81 | 208 *USt* |
| Ünal et al. (2017) | **AI:** questionnaire  **AT:** adapted from Borg and Al-Busaidi (2012)  **AP:** assess LA perceptions  **AS:** inside the classroom | √ | √ |  |  |  | √ |  |  |  |  |  | √ | √ |  | α = 0. 82 | 326 *USt* |
| Larsari and Oghli (2016) | **AI:** questionnaire  **AT:** not clear  **AP:** assess LA level  **AS:** inside the classroom |  | √ |  | √ |  |  |  |  |  |  |  |  |  |  | α = 0.78 | 90 *USt* |
| Gholami (2016) | **AI:** questionnaire  **AT:** adapted from Moini and Asadi Sajed (2012), Hashemian and Soureshjani (2011), Nematipour (2012), Rahnama and Zafarghandi (2013), Maftoon, Daftarifard and Lavasani (2011)  **AP:** assess LA level  **AS:** in/outside the classroom |  |  | √ | √ |  | √ | √ | √ |  |  |  |  | √ |  | / | 49 *USt* |
| Ceylan (2015) | **AI:** questionnaire  **AT:** existing questionnaire from Karabiyik (2008)  **AP:** assess LA level  **AS:** inside the classroom |  | √ |  |  |  |  |  | √ |  |  |  |  |  |  | α = 0.888 | 150 *USt* |
